# Effect of doped H, Br, Cu, Kr, Ge, As and Fe on structural features and bandgap of poly C13H8OS-X: a DFT calculation

**DOI:** 10.1080/15685551.2021.1877431

**Published:** 2021-02-02

**Authors:** Trung Vu Quoc, La Trieu Duong, Van Duong Quoc, Tuan Tran Quoc, Dung Nguyen Trong, Stefan Talu

**Affiliations:** a Faculty of Chemistry, Hanoi National University of Education, Hanoi, Vietnam; b Hanoi - Amsterdam High School for the Gifted, Hanoi, Vietnam; c Faculty of Physics, Hanoi National University of Education, Hanoi, Vietnam; d Faculty of Basic Science, University of Transport Technology, Hanoi, Vietnam; e Hanoi National University of Education, Faculty of Physics, Hanoi, Vietnam; f The Directorate of Research, Development and Innovation Management (DMCDI), Technical University of Cluj-Napoca, Cluj-Napoca, Cluj County, Romania

**Keywords:** Band gap, dft, doped, lattice constant

## Abstract

Structural features such as the shape, the lattice constant, the bond length, the total energy per cell, and the energy bandgap (E_g_) of C_13_H_8_OS-X are studied by the calculating Partial Density Of States (PDOS), and DOS package of the Material Studio (MS) software. Calculations show that the bond length and the bond angle between atoms insignificant change as 1.316 Å to 1.514 Å for C-C, 1.211 Å for C-O, 1.077 Å to 1.105 Å for C-H; bond angle of round one changes from 118.883° to 121.107° for C-C-C, from 117.199° to 122.635° for H-C-C, from 119.554° to 123.147° for C-C-O and from 109.956° to 117.537° for C-C-H. When C13H8OS-X doped in the order of -Br, -Cu, -Kr, -Ge, -As, and -Fe then bond lengths, bond angles between atoms have a nearly constant value. Particularly for links C-X, there is a huge change in value, respectively 1.876, 1.909, 10.675, 2.025, 2.016, 2.014 Å; the total energy change from E_tot_ = −121,794 eV to E_tot_ = −202,859 eV, and the energy band gap decreases from E_g_ = 2.001 eV to E_g_ = 0.915 eV. The obtained results are useful and serve as a basis for future experimental research.

## 1. Introduction

Polythiophenes are polymer conjugate materials that have been studied and used in many devices such as transistors, opto-electromagnetism, communication equipment, chemical/biological sensors [1–8], light-emitting diodes [9,10], photoelectric cells [11], photoelectron equipment [12,13], water-soluble sensors for detecting DNA, proteins and metal ions [14–16], thermal, optical and biological pigments [17–19] with structural, optical and electrochemical flexibility [20–25].

The reason is that these polymers can assemble spontaneously through intermolecular bonds under the action of a suitable solvent or medium [26–32]. On the other hand, Cui et Kertesz [33] showed the existence of helix polythiophene by the semi-experimental method [33] and other scientific groups showed the existence of polymeric helix [34].

One group of researchers [35–37] has found a polymeric helix structure after the polythiophenes completed bonding with the client molecules and polymers. Other groups of researchers also suggested that non-ionic polythiophenes could fold in hydrophobic solvents [38,39]. These results are determined through Scanning Electron Microscopy (SEM) but they did not observe the diversity of materials [40].

Also, when studying the effect of doped or solvent on the bond lengths of polymer materials, it cannot be studied by X-ray diffraction method or SEM method [41,42].

In recent years, several researchers have used the Density Function Theory (DFT) method to study the structure, electronic structural properties, and transition temperature of conjugated polythiophene derivatives of optical active conjugate polymers [43–47]. Besides that, we have successfully studied the effects of temperature, pressure, atoms number, annealing time on the structure of Al metal [48,49], alloys AlNi [50], NiCu [51], FeNi [52], NiAu [53], polyethylene [54], electronic structure of AuCu [55] and polymers by using DFT method to control band gap by replacing doped -S atoms with -Se atoms [56] or replacing -H atoms with -CH_3_, -NH_2_, -NO_2_ and -Cl [49,57–61] and 4 H-xiclopenta [2,1-b,3; 4-b′] or replacing dithiophene S-oxide with derivatives -O, -S, S = O, -BH_2_, -SiH_2_ [47,62–64]. Recently, we have used the DFT method to study the effects of doped groups on the electrical structure and phase transition temperature of monothiophene C_13_H_8_OS-X (X = -H, -OH, -Br, -OC_2_H_5_, -OCH_3_). The length of bond C-H = 1.09 Å in C_13_H_8_OS-H; the length of bond C-Br = 1.93 Å in C_13_H_8_OS-Br; the length of bond C-O = 1.45 Å, the length of bond C-C = 1.51 Å and the length of bond C-H = 1.10 Å in C_13_H_8_OS-OC_2_H_5_; the length of bond C-O = 1.44 Å and the length of bond O-H = 1.10 Å in C_13_H_8_OS-OCH_3_. The bond angle is 120° for C-C-C, 120° for H-C-C, 120° for C-C-O, 114° for C-S-C, and 109° for S-C-C. Similarly, the bandgap E_g_ of C_13_H_8_OS decreases to E_g_ = 1.621 eV by doped -Br and increases to 1.646, 1.697, 2.04, and 1.920 eV by replacing with impurities -H, -OH, -OC_2_H_5_ or -OCH_3_. The obtained results show that substituents have a significant influence on the molecular shape, the bond length as well as the frequency range of polythiophene derivatives [57,64].

In this article, we continue to doped atoms of cycle 4 including -H, -Br, -Cu, -Kr, -Ge, -As, and -Fe with the desire to synthesize new polythiophenes with the ability to improve their treatment, environmental stability, and electrical properties.

## 2. Method of calculation

**Figure 1.**
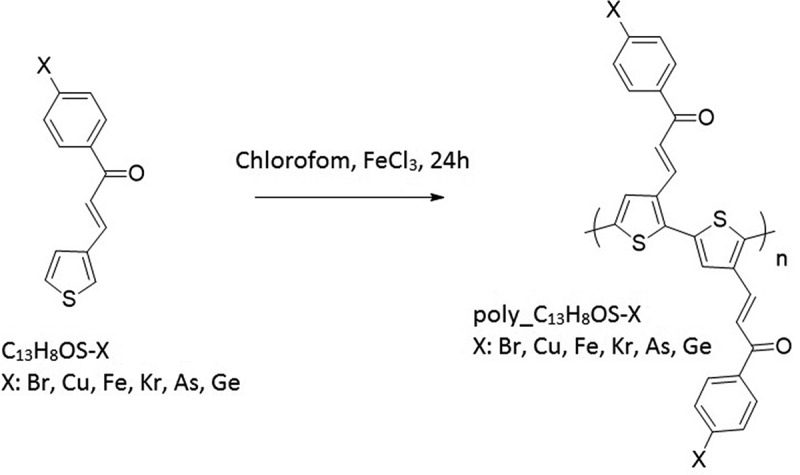
The synthetic procedure of poly (C_13_H_8_OS-X), X is -Br, -Cu, -Fe, -Kr, -As, -Ge

Figure 1 shows the synthesizing process of poly (C_13_H_8_OS-X) (X = -H, -Br, -Cu, -Kr, -Ge, -As, -Fe). The structural and electronic structural properties of poly[3-(3-phenyl prop-1-ene-3-one-1-yl) thiophene] by the calculating Partial Density Of States (PDOS), DOS package of the Material Studio (MS) software, and their transition temperatures were simulated using DFT [65–68] in the framework of DMol3 module [67] in the copyrighted Material Studio software, installed at the Center for Computational Science of the Hanoi National University of Education HNUE (Hanoi, Vietnam). The Generalized Gradient Approximation (GGA) package [69] with the PW91 parametrization for the exchange-correlation function [70,71] and the Monkhorst-Pack [72] k-point sampling were applied into a three-dimensional (3-D) unit cell of poly C_13_H_8_OS-H, C_13_H_8_OS-Br, C_13_H_8_OS-Cu, C_13_H_8_OS-Kr, C_13_H_8_OS-Ge, C_13_H_8_OS-As, C_13_H_8_OS-Fe with the lattice constants *a* = 27 Å, *b* = 13 Å, *c* = 6 Å, and the bond angles *α* = *β* = *γ* = 90°. The electron-electrons interaction was described by the Density Function Semi-core Pseudo-Potential [73] and to be considered as a homogeneous electron gas. The tolerance for energy was set at 1 × 10^−6^ eV, the displacement during the geometry optimization is at level 1 × 10^−5^ Ha/integer and 5 × 10^−3^ Å. The synthesis procedure of poly (C_13_H_8_OS-X) was shown in Figure 1.

To study the structural features and the bandgap of C_13_H_8_OS-X, we use simulations based on the Density Functional Theory (DFT) basis [74,75] included the Schrodinger model [76,77], the Hartree-Fock model [78,79], the Thomas-Fermi model [76], the Hohenberg theorem [76,80,81], and traditional Kohn-Sham theory [76,80,82]. We apply the General Gradient Approximation (GGA) [83], the Korringa-Kohn-Rostocker (KKR) [84], and the Linear-Muffin-Tin-Orbital (LMTO) [85] methods to evaluate calculations.

## 3. Results and discussion

### 3.1 Physical properties of C_13_H_8_OS-H calculated by molecular dynamics calculations

#### 3.1.1 Structural property of C_13_H_8_OS-H

**Figure 2.**
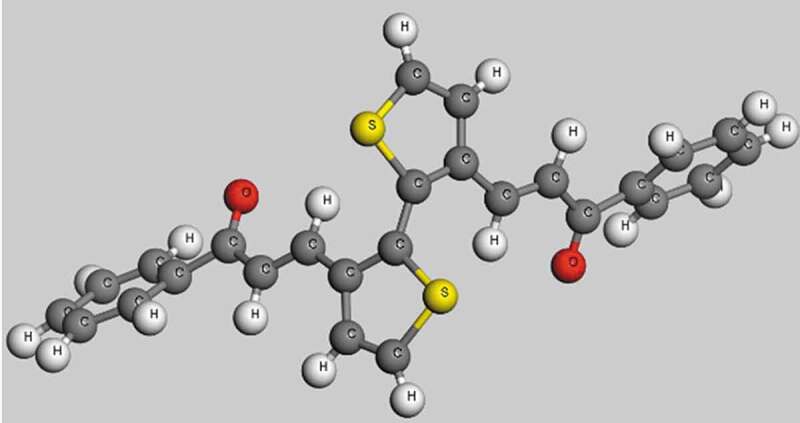
The optimized structures of poly (C_13_H_8_OS-H)_2_ calculated by molecular dynamics calculations

The optimized structures of poly (C_13_H_8_OS-H)_2_ are shown in Figure 2.

The stable structure of C_13_H_8_OS-H after running the NVE (Figure 2). The final shape of C_13_H_8_OS-H has C_13_H_8_OS-H poly structures with all C, H, S, O atoms are distributed in a unit cell of the triclinic system with the corresponding cell sizes a = 27.0951 Å, b = 11.5351 Å, c = 6.1176 Å, α = β = 90° and γ = 94.42°. The distance between atoms in round one changes from 1.380 Å to 1.41 Å for C-C, and from 1.060 Å to 1.119 Å for C-H. The distance between round one and round two changes from 1.316 Å to 1.514 Å for C-C, 1.211 Å for C-O, and from 1.077 Å to 1.105 Å for C-H. The obtained results are in good agreement with the structural determination [57,64] for which C-C = 1.33 Å, C-O = 1.23 Å, and previous calculation [57,64]. The bond length in round two changes from 1.372 Å to 1.399 Å for C-C, 1.722 Å for C-S, and from 1.106 Å to 1.148 Å for C-H. The bond angle of round one changes from 118.883° to 121.107° for C-C-C, from 117.199° to 122.635° for H-C-C, from 119.554° to 123.147° for C-C-O, and from 109.956° to 117.537° for C-C-H. The bond angle of round two changes from 111.186° to 115.008° for C-C-C, from 122.321° to 124.347° for H-C-C, 89.695° for C-S-C, from 109.929° to 114.042° for S-C-C, and 122.983° for S-C-H.

#### 3.1.2 Electronic Structure of C_13_H_8_OS-H

**Figure 3.**
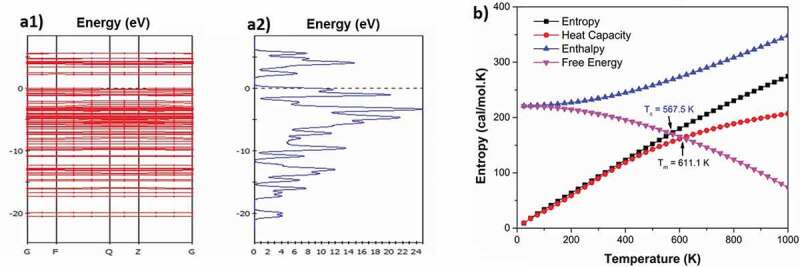
Band structures (a1), Density of states (a2); Phase transition temperature (b) zone for C_13_H_8_OS-H

The bandgap is E_g_ = 2.255 eV (Figure 3a1) and the density of electrons of C_13_H_8_OS-H (Figure 3a2) has a maximum value of 23.5%. The density of electrons for C_13_H_8_OS-H is shown in Table 1.

**Table 1.** The density of electrons for C_13_H_8_OS–H

**Energy levels (eV)**	−20	−15	−10	−5	−2.5	0	2.5	5	Result
**Density of electrons (%)**	2.334	6.625	9.423	19.462	8.925	4.011	2.629	4.151	Simulation [57,64]
	4.099	3.110	5.631	16.394	5.606	9.703	6.044	5.684	Calculation

The results of Figure 3b show that the phase transition temperature zone of C_13_H_8_OS-H ranges from 567.5 K to 611.1 K, where the glass temperature T_g_ = 567.5 K and melting temperature T_m_ = 611.1 K. These values are larger than values obtained in [57,64]. The reason for this phenomenon is that we put the material into a force field with boundary conditions different from initial boundary conditions. The obtained density of electrons for C_13_H_8_OS-H is shown in Table 1.

According to Table 1, when the energy levels are −20, – 15, – 10, −5, −2.5, 0, 2.5, and 5 eV, the corresponding electron densities of C13H8OS-H are 4.099 %, 3.110 %; 5.631 %, 16.394 %, 5.606 %, 9.703 %, 6.9044 %, and 5.684%.

### 3.2. The effect of doped on structural features of C_13_H_8_OS-X

**Figure 4.**
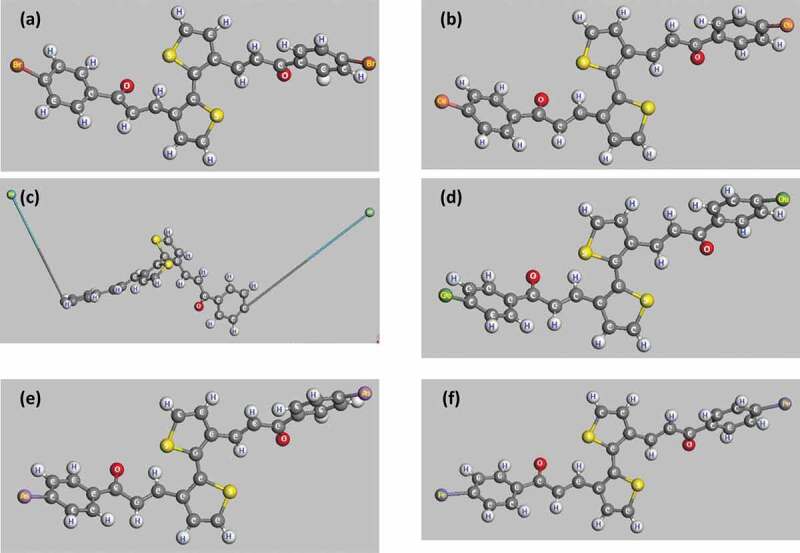
Optimized structures of Br-doped (a), Cu-doped (b), Kr-doped (c), Ge-doped (d), As-doped (e) and Fe-doped (f) C_13_H_8_OS-X models

The obtained results show that the distance between the atoms and the bond angle does not change significantly, and that is completely consistent with previous simulations [57,64]. However, the distance between the doped atoms changes strongly for poly(C_13_H_8_OS-H) doped. Concretely this distance is 1.116 Å for doped C-H (Figure 4a); 1.876 Å for doped C-Br (Figure 4b); 1.909 Å for doped C-Cu (Figure 4c); 10.675 Å for doped C-Kr (Figure 4d); 2.025 Å for doped C-Ge (Figure 4e); 2.016 Å for doped C-As (Figure 4f); 2.014 Å for doped C-Fe (Figure 4f). Structural features of pure and doped C_13_H_8_OS-X, X = H, Br, Cu, Kr, Ge, As, Fe are shown in Table 2, respectively.

**Table 2.** Structural features of pure and doped C_13_H_8_OS-X, X = H, Br, Cu, Kr, Ge, As, Fe

Model	Doped element	C-X bond lengths (Å)
PTH	H	1.116
PTB	Br	1.876
PTC	Cu	1.909
PTK	Kr	10.675
PTG	Ge	2.025
PTA	As	2.016
PTF	Fe	2.014

### 3.3. The effect of doped on electronic structures of C_13_H_8_OS-X

**Figure 5.**
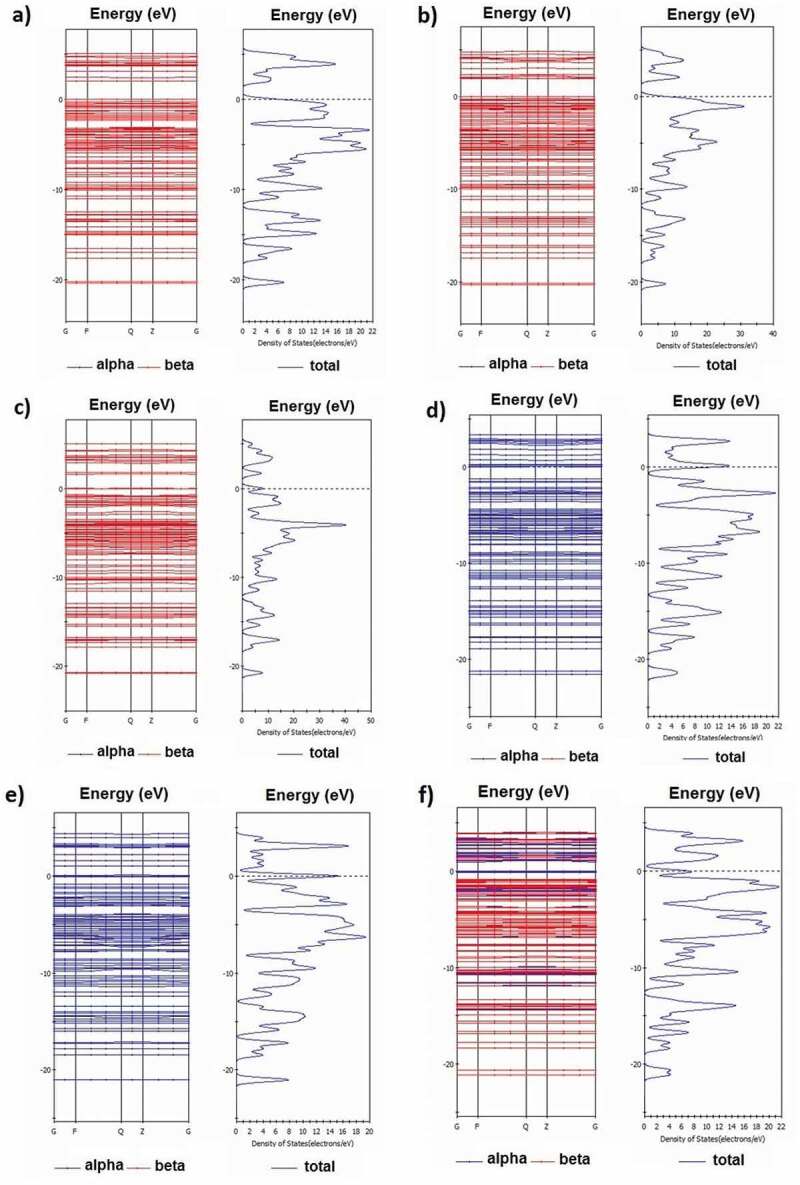
Band structure and density of states of Br-doped (a), Cu-doped (b), Kr-doped (c), Ge-doped (d), As-doped (e), and Fe-doped (f) with C_13_H_8_OS-X

**Figure 6.**
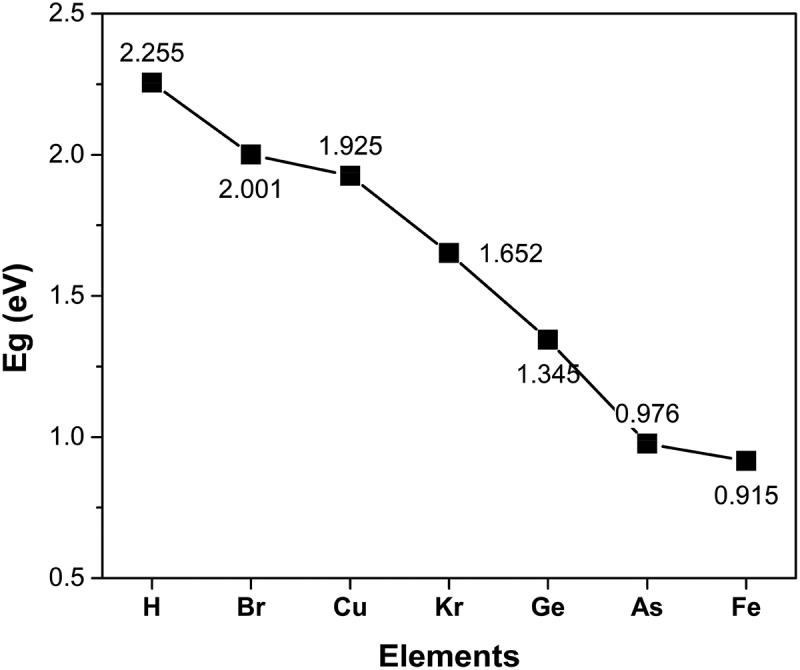
Bandgap values of metal-doped C_13_H_8_OS-X models with different elements

**Figure 7.**
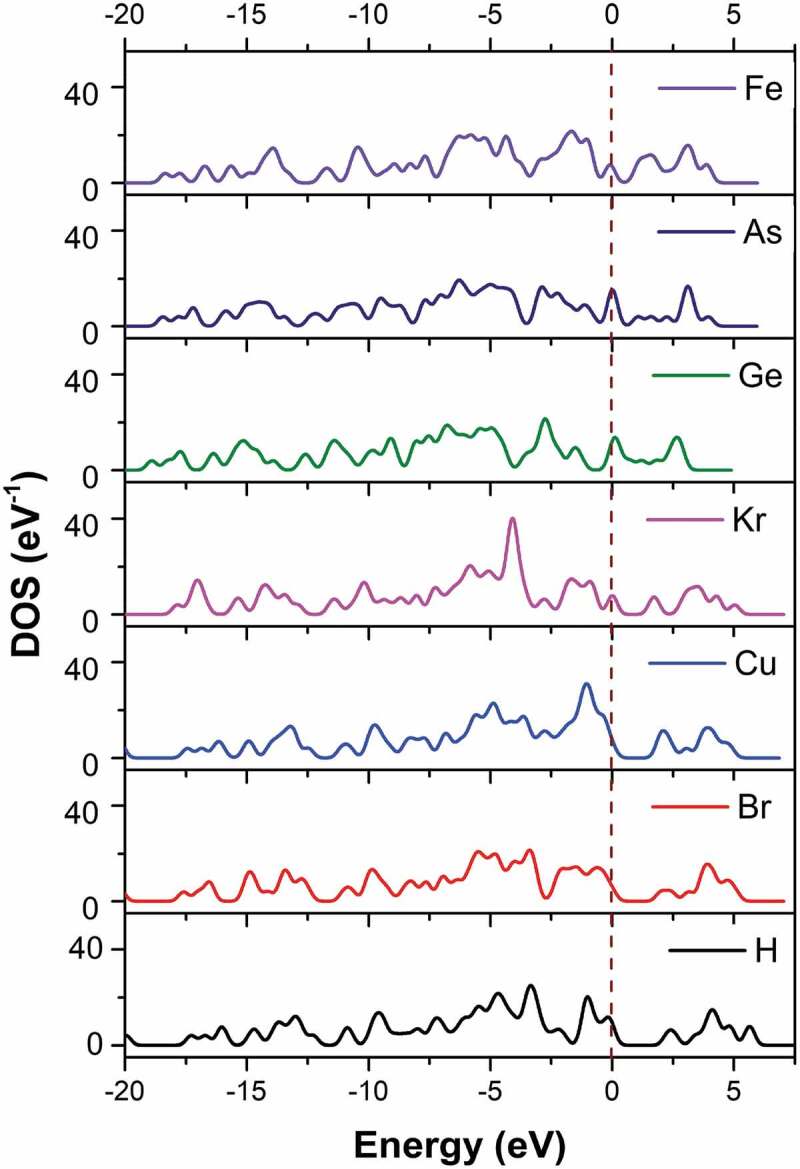
The density of states of metal-doped C_13_H_8_OS-X models with different elements

Table 3 gives the valence electrons and the value of bandgap energy for doped C_13_H_8_OS-X. The bandgap energy for C_13_H_8_OS-X doped -H, -Br, -Cu, -Kr, -Ge, -As, -Fe decreases. The values of bandgap energy for doped C_13_H_8_OS-X are shown in Figure 6. The band structure and the density of states for C_13_H_8_OS-X doped -H, -Br, -Cu, -Kr, -Ge, -As, -Fe are shown in Figure 5, Figure 7 describes the density of states for metal-doped C_13_H_8_OS-X models with different elements. The obtained results show that the molecules have the shape of box with precise cell sizes a = 27.095 Å, b = 11.535 Å, c = 6.118 Å for C_13_H_8_OS-H; a = 25.305 Å, b = 12.398 Å, c = 6.070 Å for C_13_H_8_OS-Br; a = 25.678 Å, b = 13.049 Å, c = 5.928 Å for C_13_H_8_OS-Cu a = 26.092 Å, b = 12.970 Å, c = 6.064 Å C_13_H_8_OS-Kr; a = 26.160 Å, b = 12.799 Å, c = 6.146 Å for C_13_H_8_OS-Ge; a = 26.348 Å, b = 12.913 Å, c = 6.112 Å for C13H8OS-As and a = 24.400 Å, b = 13.662 Å, c = 5.993 Å for C_13_H_8_OS-Fe. The bond angles of different poly(C_13_H_8_OS-X) derivatives are 123.017° with C-C-H of C_13_H_8_OS-H; 115.798° with C-C-Br of C_13_H_8_OS-Br; 120.957° with C-C-Cu of C_13_H_8_OS-Cu; 123.017° with C-C-Br of C_13_H_8_OS-Kr; 120.617° with C-C-Ge of C_13_H_8_OS-Ge; 125.009° with C-C-As of C_13_H_8_OS-As and 11.022° with C-C-Fe of C_13_H_8_OS-Fe. When the energy levels are −20, −15, −10, −7.5, −5, 0, 5 and 7.5 eV, the corresponding densities of electrons for C_13_H_8_OS-H are 4.099 %, 3.110 %, 5.631 %, 16.394 %, 5.606 %, 9.703 %, 6.044 % and 5.684 %.

**Table 3.** Bandgap values of pure and doped C_13_H_8_OS-X

Model	Doped element	Valence electrons of doped elements	Estimated band gap energy (eV)	Total energy per cell (eV)
PTH	H	1s^1^	2.255	−53,019
PTB	Br	3d^10^ 4s^2^ 4p^5^	2.001	−193,091
PTC	Cu	3d^10^ 4s^1^	1.925	−142,278
PTK	Kr	3d^10^ 4s^2^ 4p^6^	1.652	−202,859
PTG	Ge	3d^10^ 4s^2^ 4p^2^	1.345	−166,018
PTA	As	3d^10^ 4s^2^ 4p^3^	0.976	−174,664
PTF	Fe	3d^6^ 4s^2^	0.915	−121,794

The density of electrons for C_13_H_8_OS-X doped the functional groups -Br, -Cu, -Kr, -Ge, -As, -Fe changes greatly. For example when the energy level at −20 eV, the density of electrons is 4.099% for C_13_H_8_OS-X doped -H, 3.262% for C_13_H_8_OS-X doped -Br,4.071% for C_13_H_8_OS-X doped -Cu, 0,014% for C_13_H_8_OS-X doped -Kr, 0% for C_13_H_8_OS-X doped -Ge and -As, 0.063% for C_13_H_8_OS-X doped – Fe. At −15 eV, the corresponding densities of electrons are 3.110%, 10.772%, 6.619%, 2.511%, 11.541%, 9.02% and 3.785%. At −10 eV, the corresponding densities of electrons are 5.631%, 11.681%, 8.704%, 10.836%, 7.388%, 3.312% and 7.529%. At-5 eV, the corresponding densities of electrons are 16.394%, 18.743%, 21.630%, 17.866%, 17.608%, 17.583% and 15.650%; At −2.5 eV, the corresponding densities of electrons are 5.606%, 3.954%, 9.327%, 3.585%, 15.764%, 12.443% and 11.425%. At 0, the corresponding densities of electrons are 9.703%, 6.343%, 7.909%, 7.806%, 12.238%, 15.230% and 7.020%. At 2.5 eV, the corresponding densities of electrons are 6.044%, 3.857%, 5.089%, 6.786%, 12.214%, 2.208% and 6.016%. At 5 eV, the corresponding densities of electrons are 5.684%, 6.493%, 3.290%, 3.903%, 0, 0 and 0. This shows that in the valence region, the density of electrons has the largest percentage extending the maximum value at the energy range of −5 eV. Also, the bandgap E_g_ decreases for the doped in the order of -Br, -Cu, -Kr, -Ge, -As, and -Fe. Besides, the total energy E_tot_ of the system decreases suddenly at -Kr, which shows that the E_tot_ of the −4p subclass of the material decreases for increasing the subclass from −4p^2^ to −4p^3^, −4p^5^, and −4p^6^. The obtained results are used as the basis for future experimental research and used to predict the structural features and electronic structure of C_13_H_8_OS-X doped.

**Figure:**
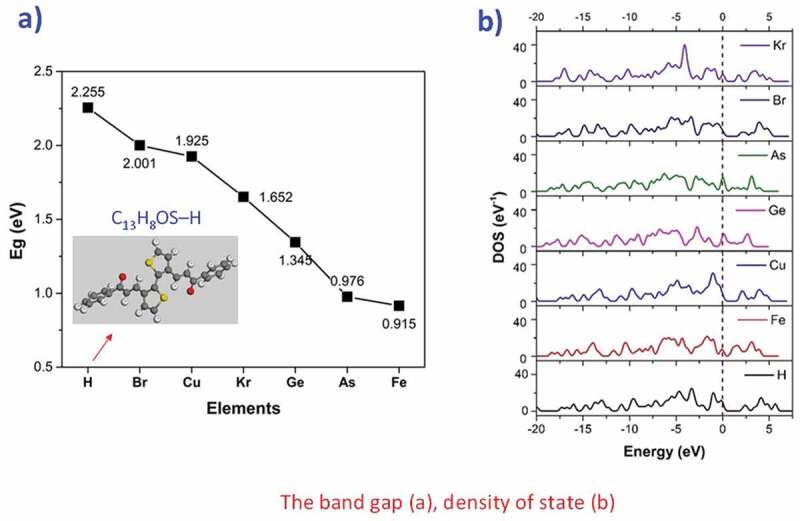

## 4. Conclusion

The effects of doped -H, -Br, -Cu, -Kr, -Ge, -As, and -Fe on structural features and the electronic structure of C_13_H_8_OS-X are studied by the GGA package PW91 of the Material Studio software copyrighted based on the DFT method. Structural features such as the lengths of the bonds C-H, C-Fe, C-Ge, C-C, C-Br, C-O, C-Cu, C-Kr, C-As and the bond angles H-C-H, H-C-C, C-C-C, C-Fe-H, C-Ge-C, C-Br-C, C-O-C, Cu-C-C, Kr-C-C, As-C-C do not change significantly. However, when then total energy change of system decrease from E_tot_ = −121,794 eV to E_tot_ = −202,859 eV; and the total energy and the bandgap of C_13_H_8_OS-X doped in the order of -H, -Br, -Cu, -Kr, -Ge, -As and -Fe decrease from E_tot_ = – 121,794 eV to E_tot_ = – 202,859 eV and from E_g_ = 2.001 eV to E_g_ = 0.915 eV respectively. This shows that the influence of benzene ring and impurities on electronic structure features and the bandgap of poly materials is important and these results are considered as a basis for future experimental research.

## Data Availability

The data that support the findings of this study are available from the corresponding author upon reasonable request.
